# Supplementary material for: Human single neuron activity precedes emergence of conscious perception
Source: Nat Commun. 2018 May 25;9:2057. doi: 10.1038/s41467-018-03749-0 (PMC5970215; doi:10.1038/s41467-018-03749-0)
Supplement: Supplementary file 1 — Supplementary Information(PDF 2907 kb) [file 41467_2018_3749_MOESM1_ESM.pdf]

Supplementary Information

# Human single neuron activity precedes emergence of conscious perception

**Gelbard-Sagiv et al.**

*Nature Communications* (2018)

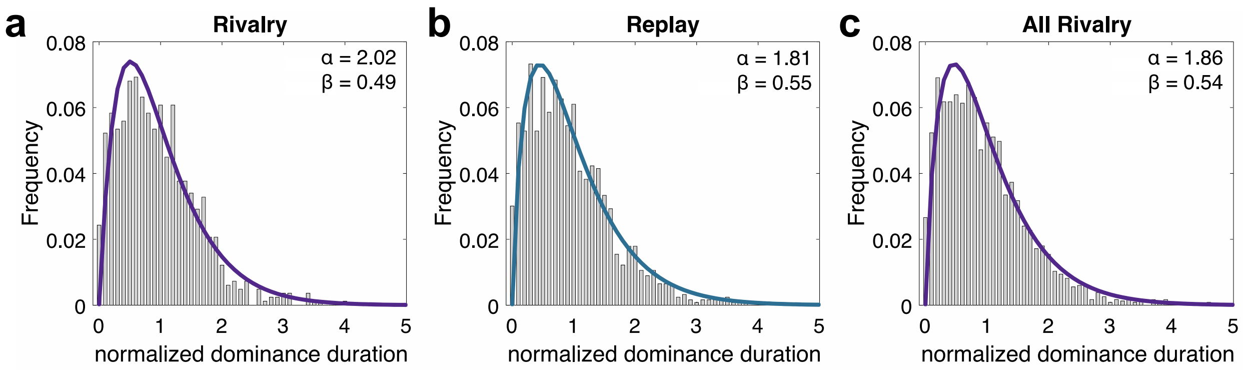


**Supplementary Figure 1 | Distribution of dominance duration.** Relative dominance duration distribution (i.e. expressed as a function of the average dominance duration for each image) and the fitted gamma function $f(x) = \frac{1}{\alpha^{\beta}\Gamma(\alpha)}x^{\alpha-1}e^{-\frac{x}{\beta}}$ ($\alpha$ = shape; $\beta$ = scale), for the rivalry blocks used to generate the replay blocks (**(a)**; only one or two of the rivalry blocks for each pair of images were followed by a matched replay block), the replay blocks **(b)** and all rivalry blocks **(c)**.

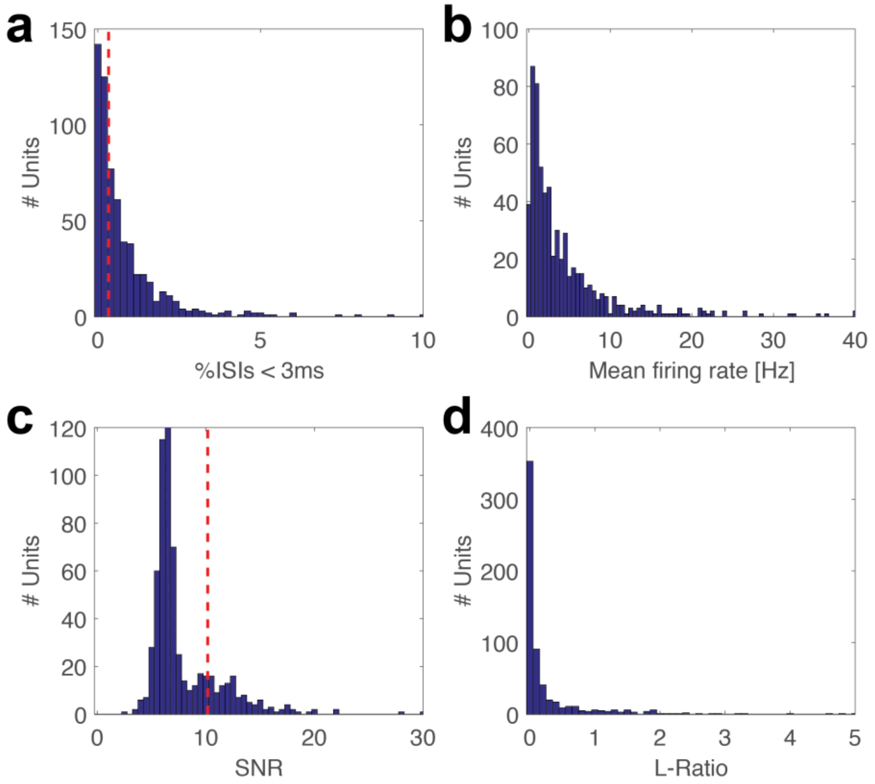


**Supplementary Figure 2 | Spike sorting assessment.** Histograms showing the distribution of proportion of inter-spike intervals shorter than 3ms **(a)**, mean firing rates **(b)**, SNR calculated as the peak to peak amplitude divided by the standard deviation of the residuals **(c)**, and L-ratio **(d)**. Red dashed lines in (a) and (c) denote the mean for the units defined as single-units.

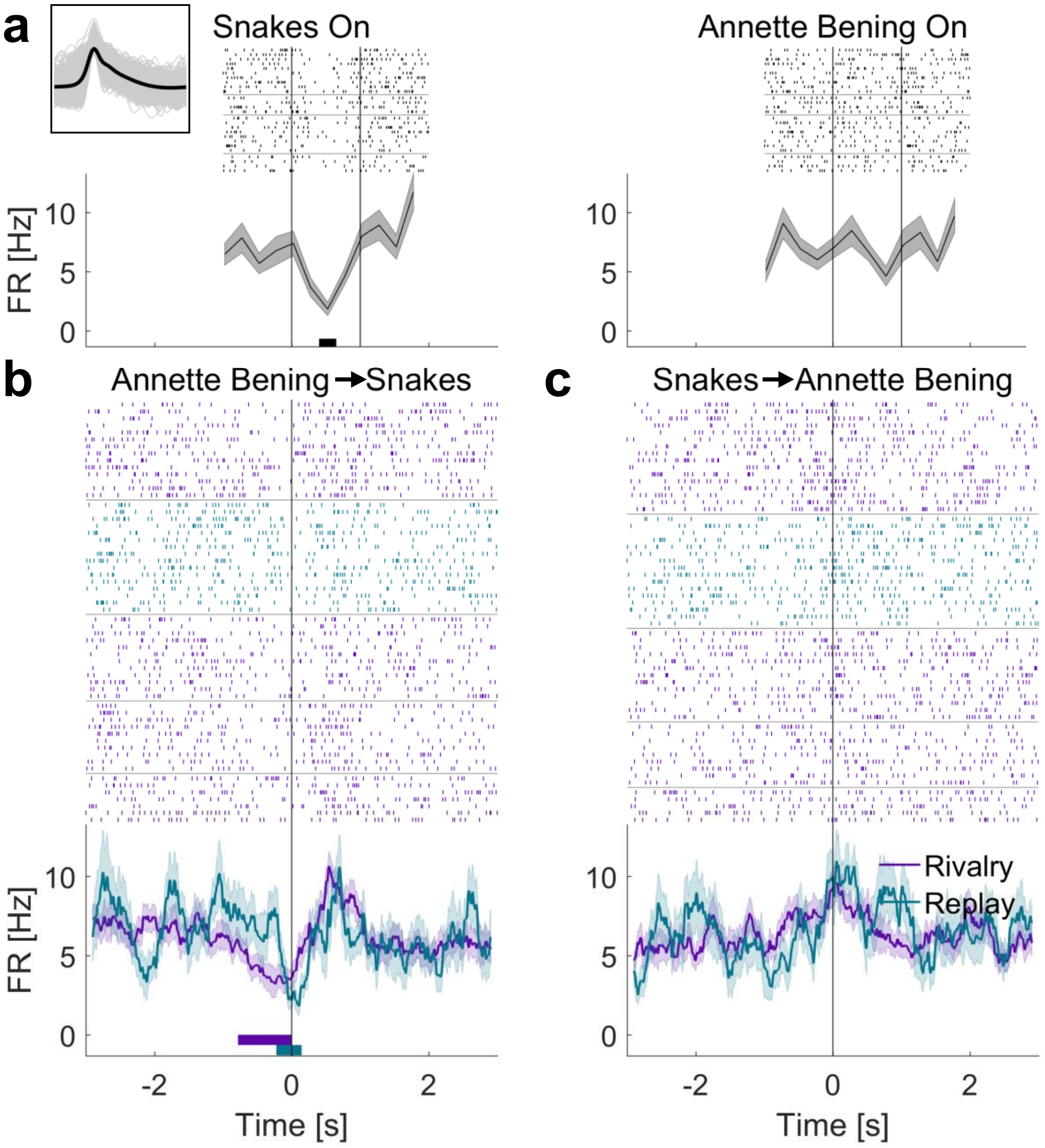


**Supplementary Figure 3 | A unit negative response pattern is reinstated during rivalry and replay.** Activity of a multi-unit in amygdala during rivalry and replay, conventions as in Fig. 2. **(a,b)** Responses to the non-rivalrous presentation of snakes (a) or the actress Annette Bening (b) images. **(c)** Neuronal firing around the report of transition onset to the snakes image (t=0; end of Annette Bening exclusive dominance) during rivalry (purple) and replay (cyan). **(d)** Neuronal firing around the report of transition onset to the Annette Bening image. Note that this unit responds to the non-rivalrous presentation of the snakes image (a) by decreasing its FR below baseline, and this response pattern is reinstated prior to the report of perceptual transition to the snakes image during rivalry and replay, but earlier in rivalry as compared to replay.

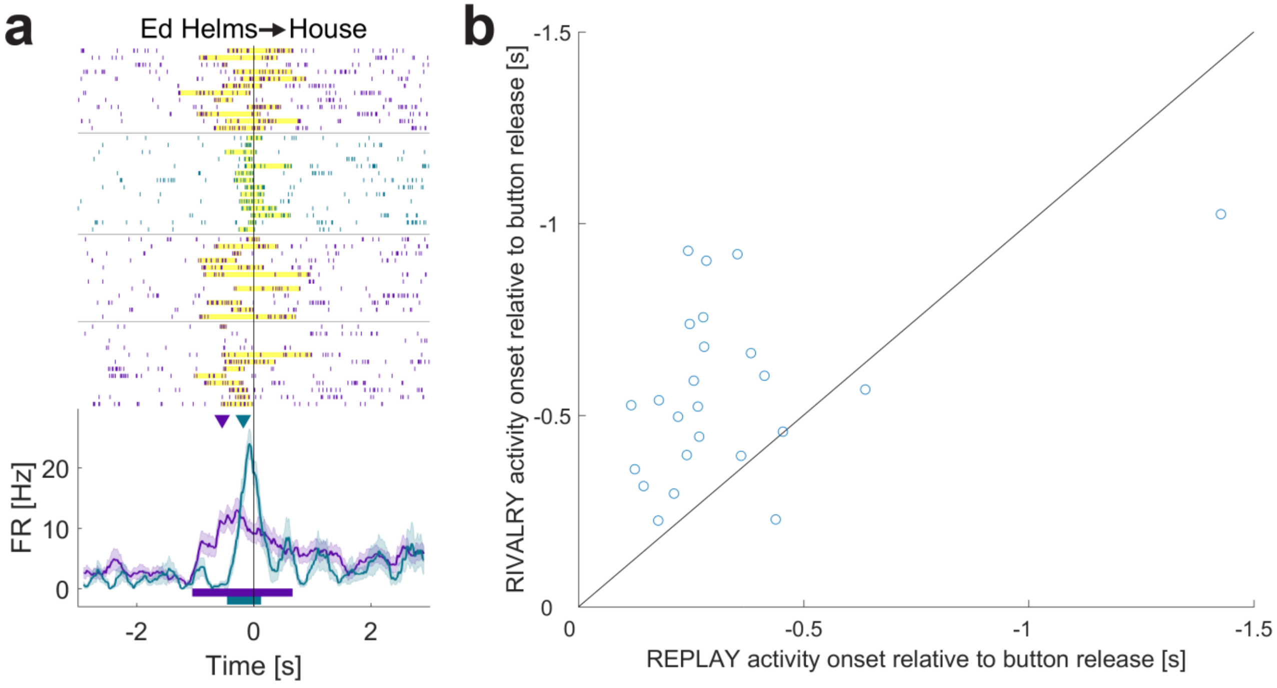


**Supplementary Figure 4 | A trial-by-trial response detection algorithm applied to MTL data replicates the effect of earlier activity onset during rivalry as compared to replay. (a)** A trial-by-trial response detection algorithm applied to the data in Fig. 2c. **(b)** Median activity onset time in rivalry vs. replay for all units for which such activity onset was detected in both conditions (n=24; Methods). Note that most of the points are above the diagonal (i.e. rivalry earlier than replay).

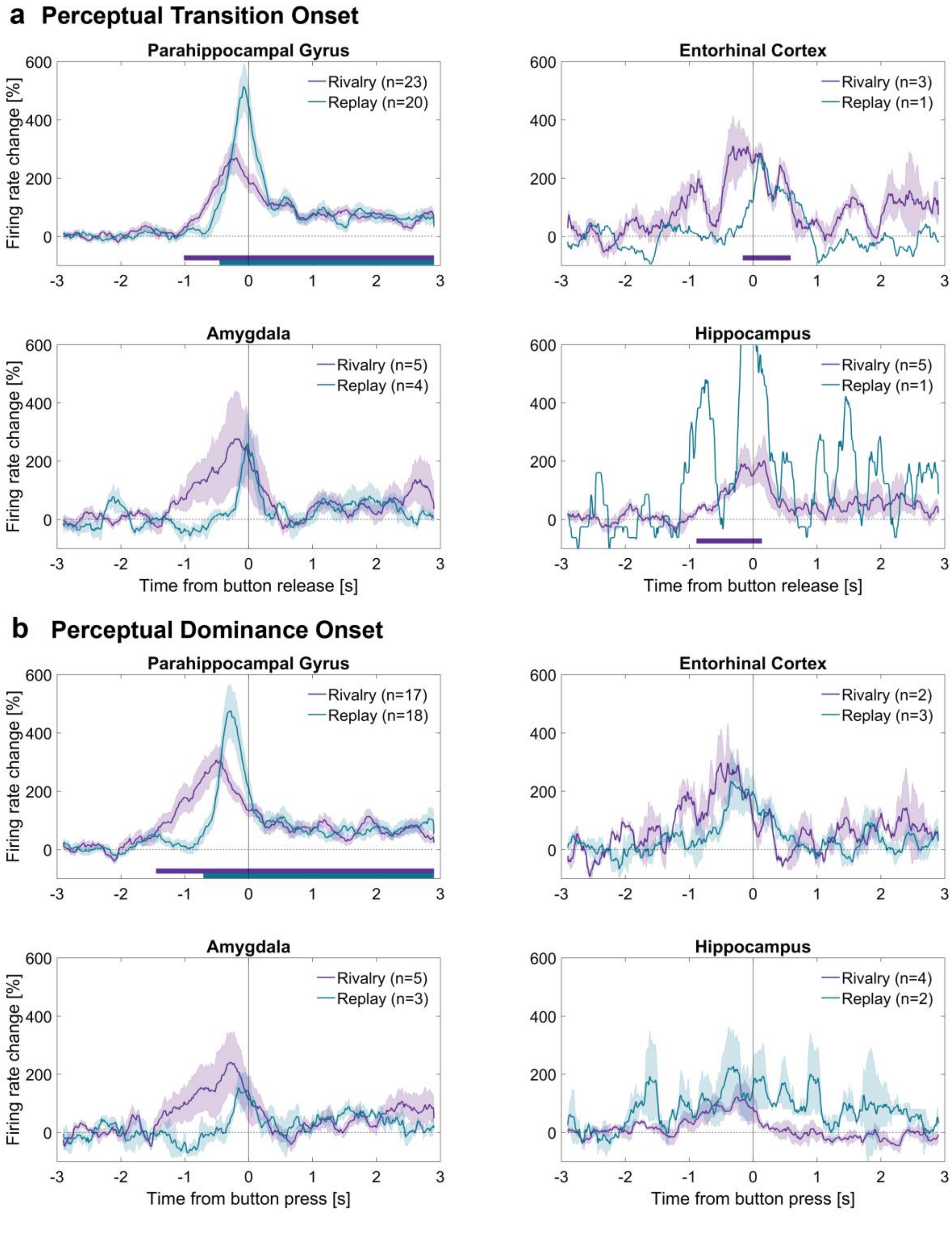


**Supplementary Figure 5 | Population-level anticipatory activity for MTL subregions.** Conventions as in Fig. 4a. **(a)** FR percent change (mean±SE) around the report of perceptual transition onset to the preferred image is averaged across all rivalry-active (purple) and replay-active traces (cyan), separately for each MTL region. **(b)** Same for perceptual dominance onset. Due to the small number of units per region, and since most patients had responsive neurons in only one or two regions we could not reliably quantify the difference between MTL subregions.

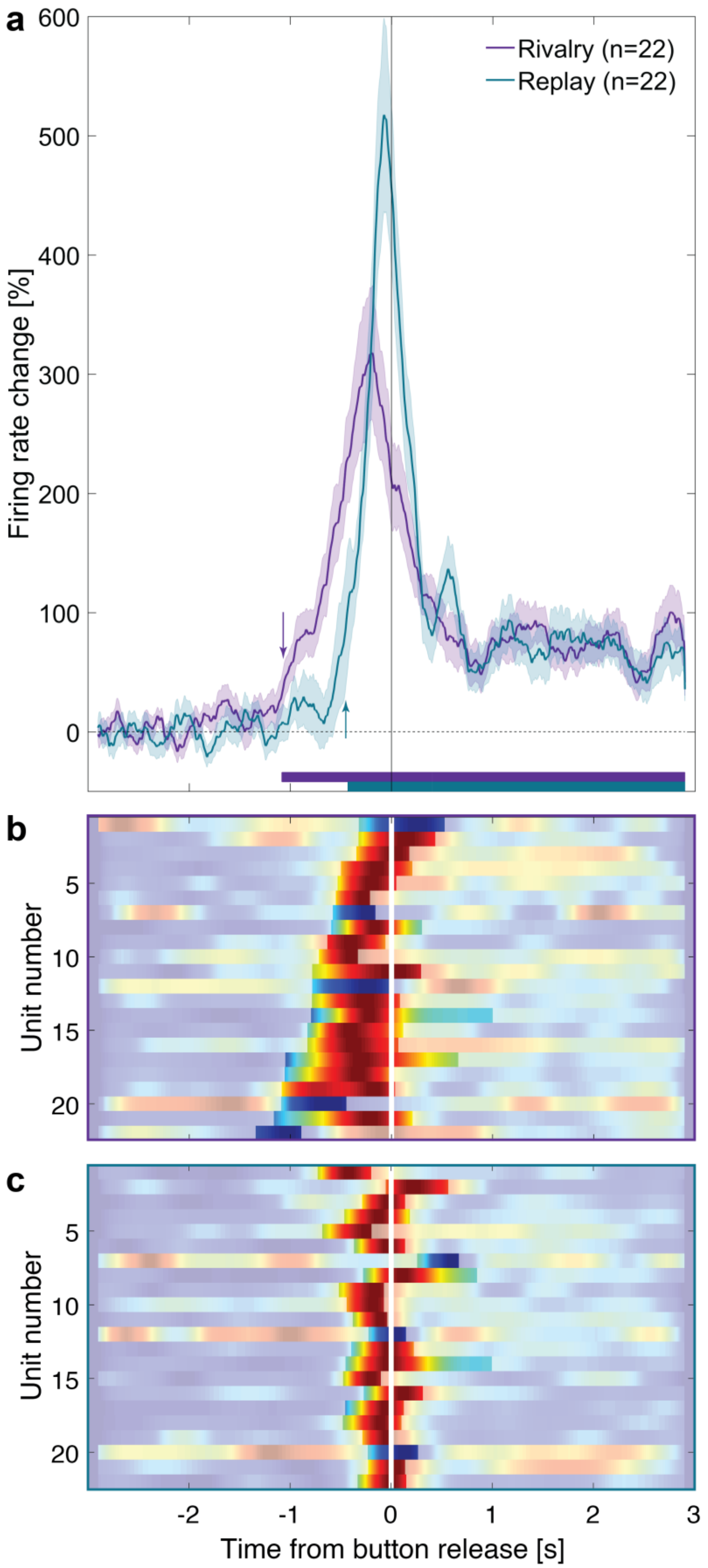


**Supplementary Figure 6 | MTL population-level activity for units that were active both during rivalry and replay.** Same as Fig. 4, but for the intersection of units that responded around the transition to their preferred image both during rivalry and replay (n=22). **(a)** FR percent change (mean±SE) around the report of perceptual transition onset to the preferred image during rivalry (purple) and replay (cyan). **(b,c)** Normalized FR time courses during rivalry (b) and replay (c) are presented in color code (dark blue=0; dark red=1), with periods of significance at the unit level highlighted. Units in (b) and (c) are ordered by their significant activity onset during rivalry.

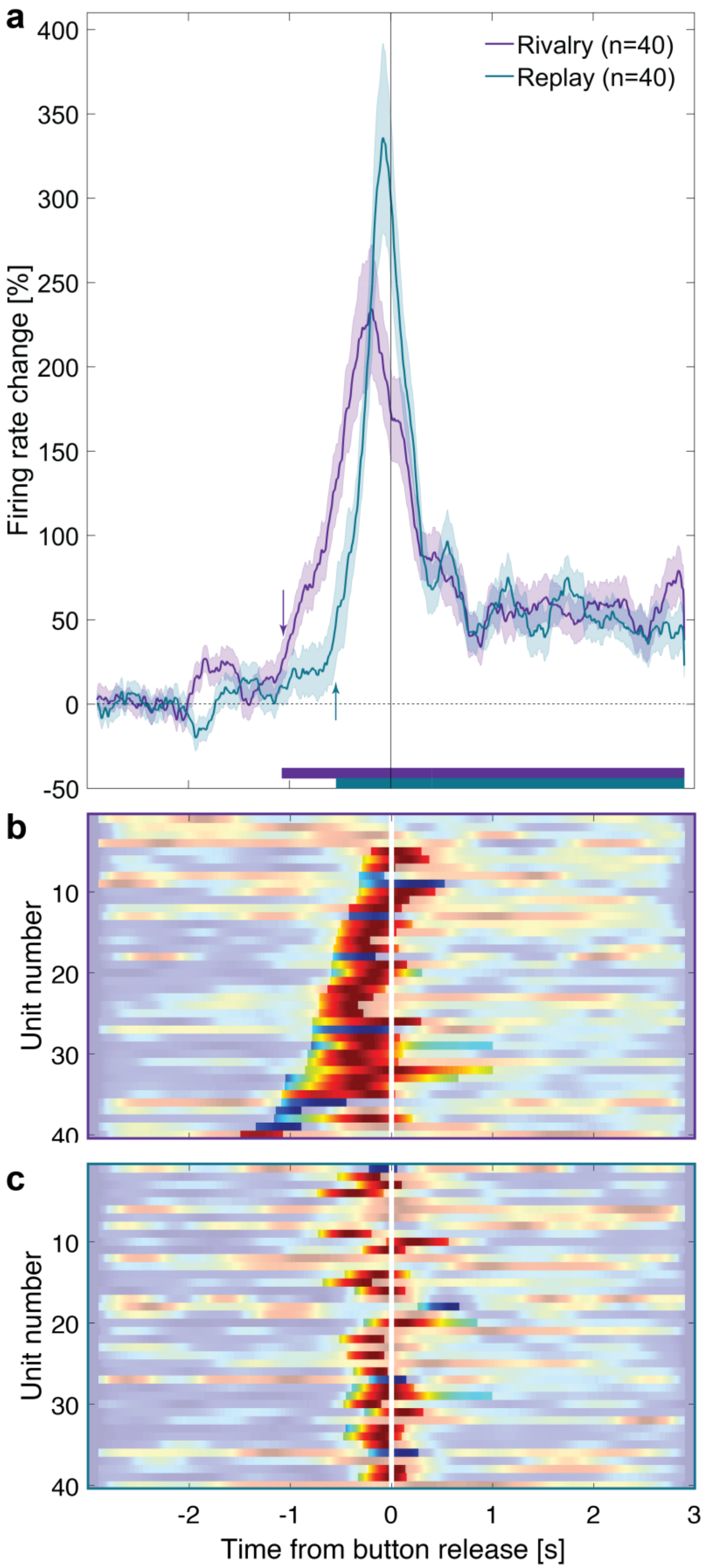


**Supplementary Figure 7 | MTL population-level anticipatory activity for union of units that were active during rivalry and/or replay.** Same as Supp. Fig. 6, but for the *union* of units that responded around the transition to their preferred image in rivalry and/or in replay (n=40). **(a)** FR percent change (mean±SE) around the report of perceptual transition onset to the preferred image during rivalry (purple) and replay (cyan). **(b,c)** Normalized FR time courses during rivalry (b) and replay (c) presented in color code. Units in (b) and (c) are ordered by their significant activity onset during rivalry. Note that many of the units that were deemed active exclusively in rivalry or in replay (i.e. highlighted only in (b) or in (c)) were actually active to some extent on the other condition, but were missed by the conservative permutation analysis.

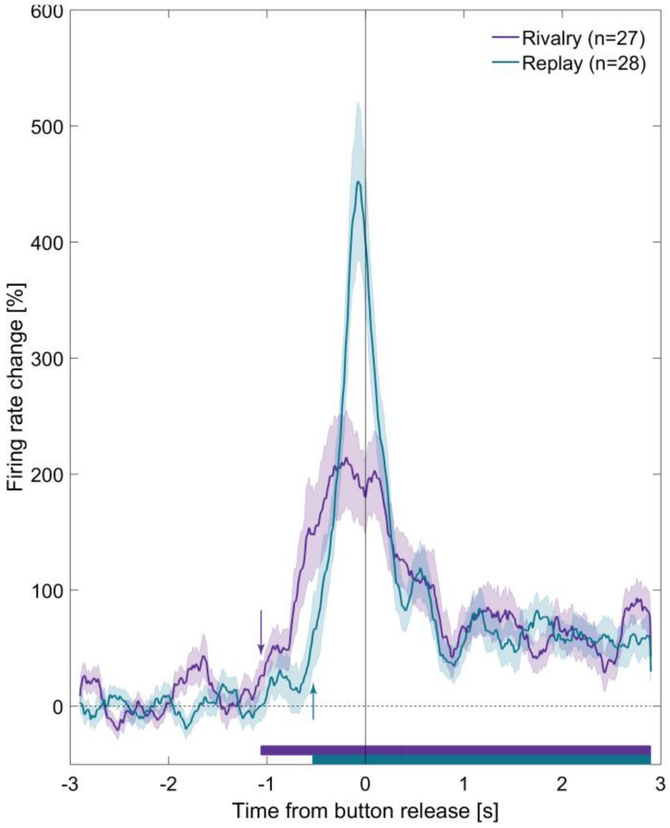


**Supplementary Figure 8 | MTL population-level anticipatory activity after equating the number of trials in rivalry and replay**. FR percent change (mean±SE) around the report of perceptual transition onset to the preferred image during rivalry (purple) and replay (cyan). Conventions as in Fig. 4a. Note that for generating this figure the individual unit activity onset analysis was rerun with the new number of trials, hence the different number of active traces as compared to Fig. 4.

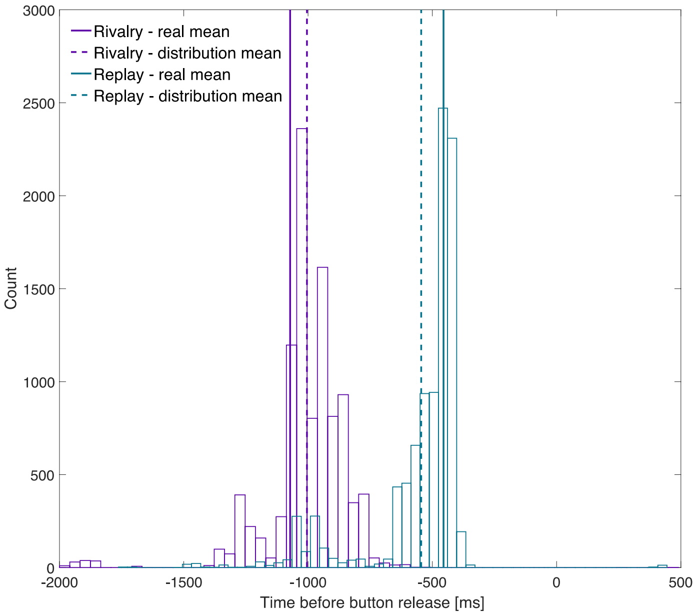


**Supplementary Figure 9 | Distributions of the bootstrapped population-level anticipatory activity onset in rivalry and replay.** Dashed lines represent the means of the bootstrapped distributions, solid line – the actual values. Both rivalry and replay actual values are at the very far end of the other condition's bootstrapped distribution ($p<0.0002$).

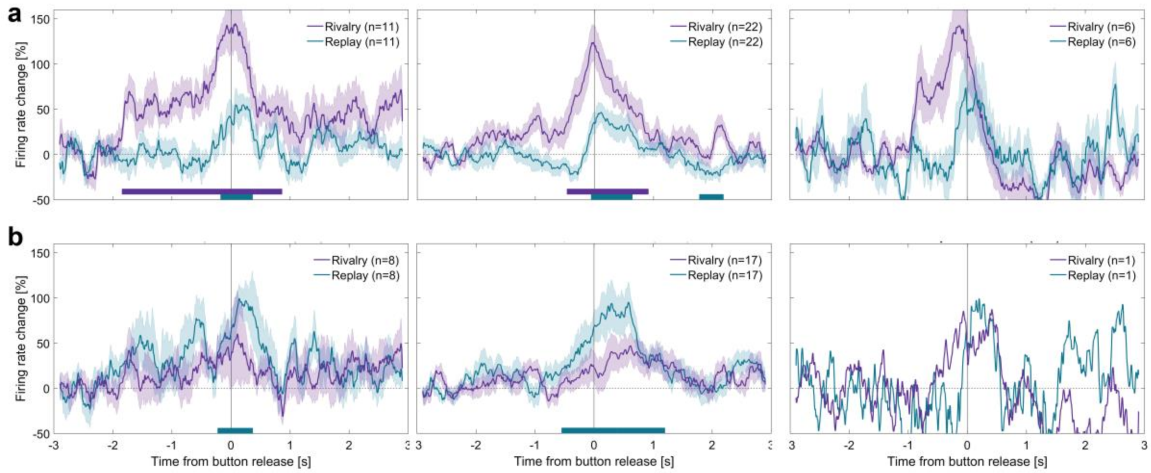


**Supplementary Figure 10 | ACC/preSMA activity profiles at the individual patient level.** FR percent change (mean±SE) around the report of perceptual transition onset during rivalry (purple) and replay (cyan), for each one of the three patients who had electrodes in these sites (each column represents data from one patient). Conventions as in Fig. 5b-c. **(a)** rivalry-active units **(b)** replay-active units. The third patient only had one such unit, hence no SE presented. The patient on the second column also had units in SMA (Fig. 6).

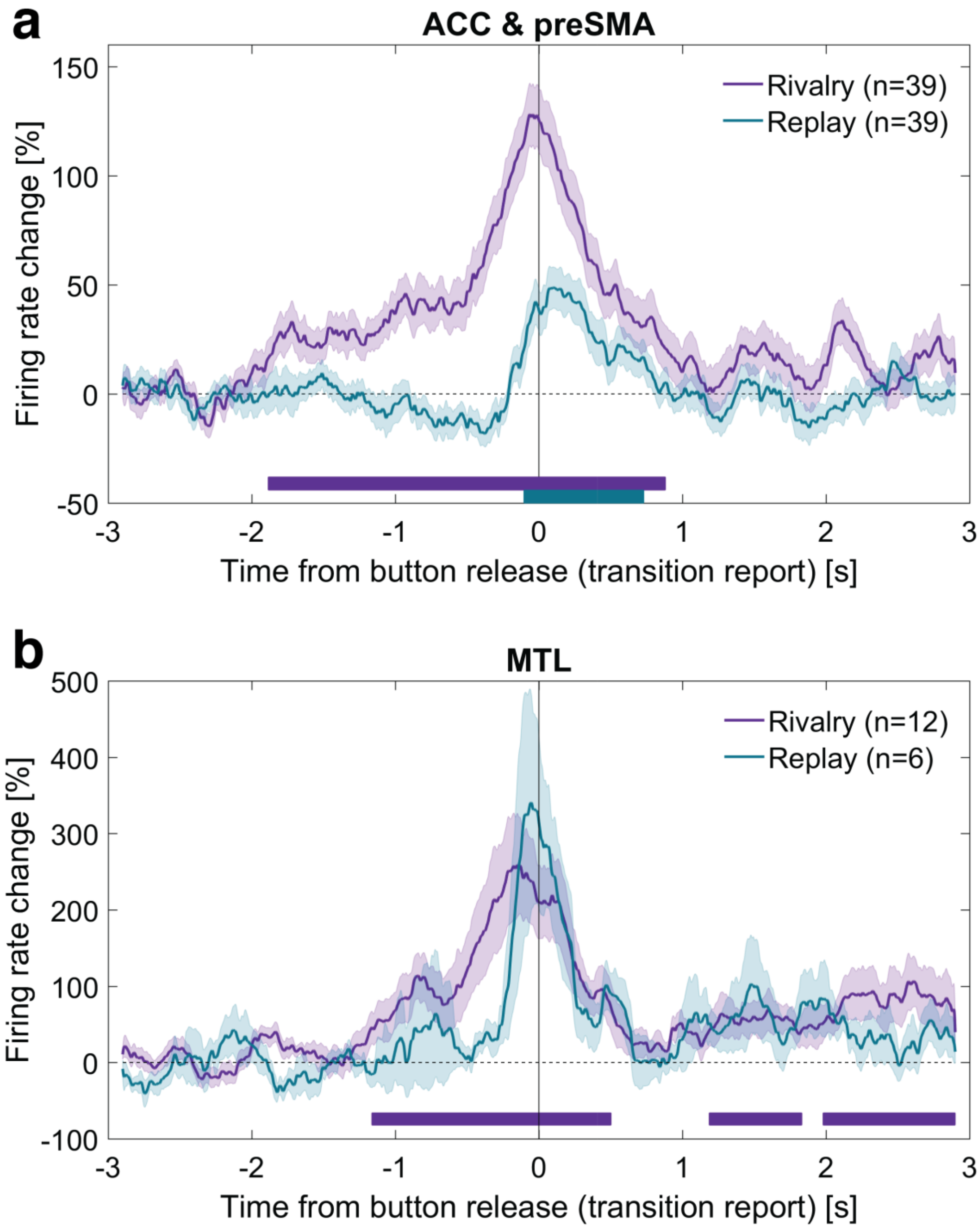


**Supplementary Figure 11 | ACC/preSMA vs. MTL activity for the subset of patients that had responsive units in both regions (n=3).** FR percent change (mean±SE) around the report of perceptual transition onset. **(a)** ACC/preSMA rivalry-responsive units during rivalry (purple) and replay (cyan). This panel is identical to Fig. 5b. **(b)** rivalry-active (purple) and replay-active (cyan) MTL traces during from the same patients. Conventions as in Fig. 4a.

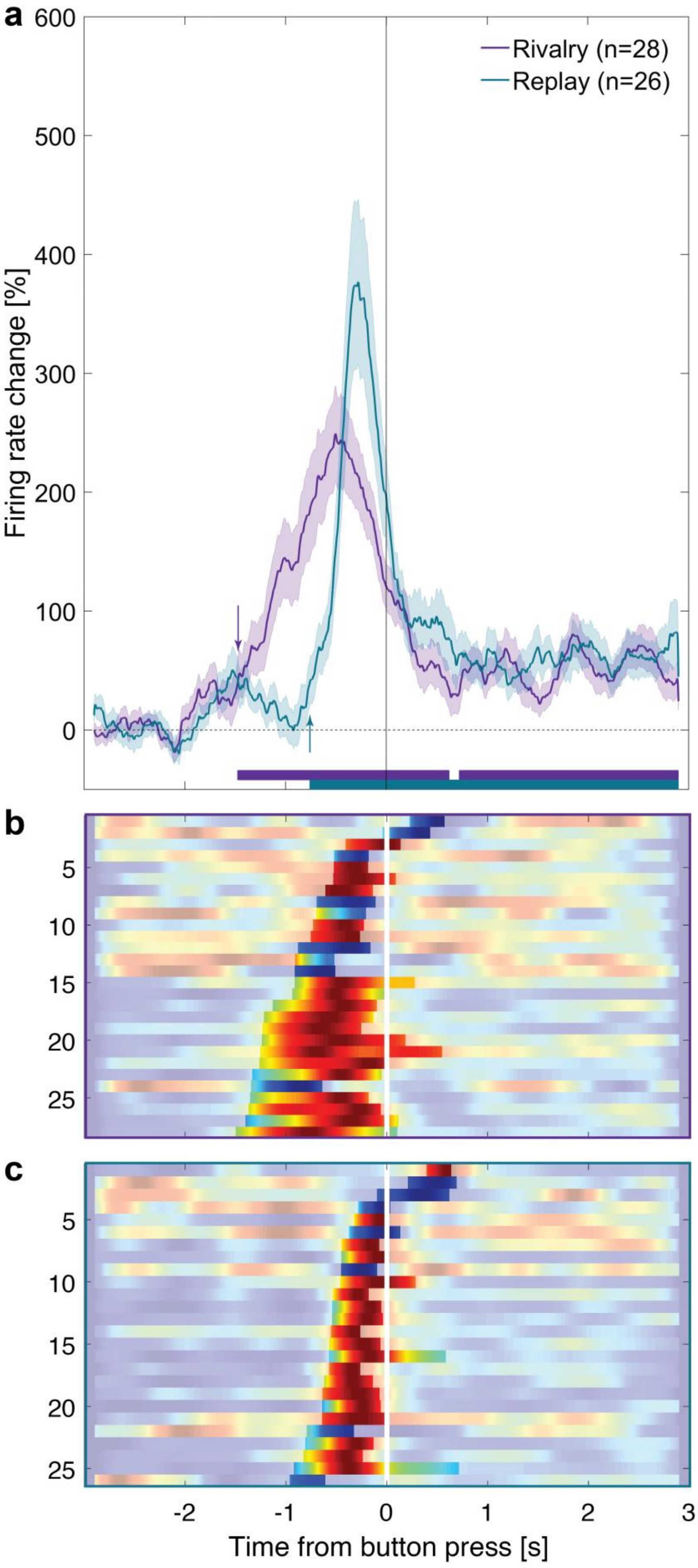


**Supplementary Figure 12 | MTL population-level anticipatory activity around dominance onset.** Same as Fig. 4 exept that here time zero represent the preferred image's dominance onset report (button press) instead of transition onset (other button release). **(a)** FR percent change (mean±SE) around the report of perceptual dominance onset of the preferred image is averaged across all MTL rivalry-active (purple; n=28) and replay-active traces (cyan; n=26). Supp. Fig. 5b present the same data for each MTL subregion separately. Normalized FR time courses for all MTL rivalry-active **(b)** and replay-active **(c)** units, with periods of significance at the unit level highlighted.

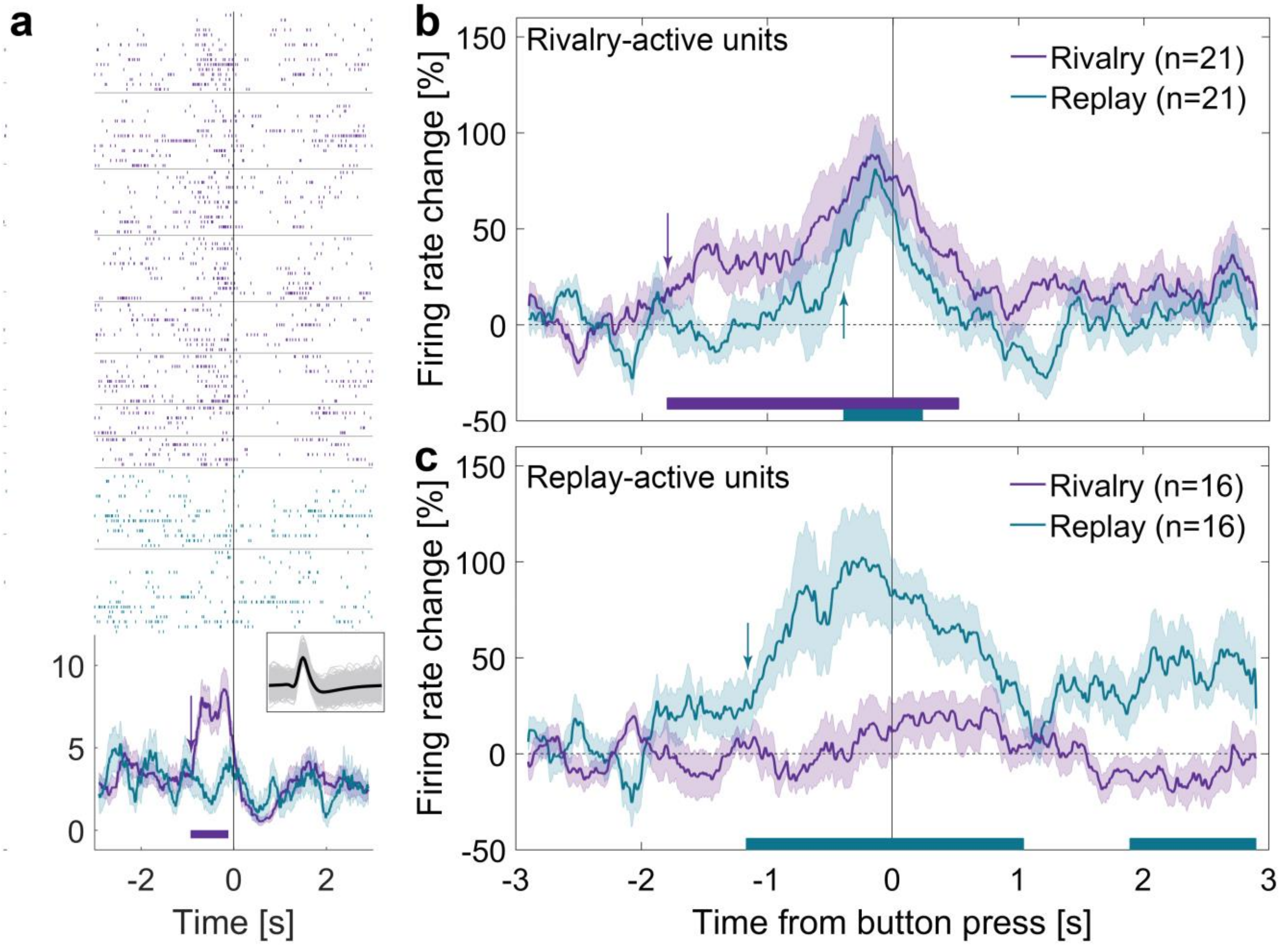


**Supplementary Figure 13 | ACC and preSMA activity around dominance onset report during rivalry and replay.** identical to Fig. 5 except that here time zero represent dominance onset report (button press) instead of transition onset. **(a)** Firing of an ACC multi-unit around the report of a perceptual transition onset during rivalry (purple) and replay (cyan). Average FR percent change (mean±SE) across all rivalry-active units **(b)** and replay-active units **(c)**.

| | | PHG | | | EC | | | AMY | | | HIPP | | | ACC | | preSMA | | SMA | |
|---|---|---|---|---|---|---|---|---|---|---|---|---|---|---|---|---|---|---|---|
| **Patient** | **Sessions** | **Units** | **NON-RI** | **RI/RE** | **Units** | **NON-RI** | **RI/RE** | **Units** | **NON-RI** | **RI/RE** | **Units** | **NON-RI** | **RI/RE** | **Units** | **RI/RE** | **Units** | **RI/RE** | **Units** | **RI/RE** |
| **1** | 1 | 0 (0) | 0 (0) | 0 (0) | 9 (3) | 1 (0) | 0 (0) | 5 (1) | 1 (0) | 0 (0) | 8 (4) | 1 (1) | 0 (0) | 0 (0) | 0 (0) | 0 (0) | 0 (0) | 0 (0) | 0 (0) |
| **2** | 2 | 22 (9) | 11 (4) | 4 (1) | 20 (11) | 2 (1) | 0 (0) | 25 (12) | 0 (0) | 0 (0) | 26 (15) | 1 (1) | 1 (1) | 0 (0) | 0 (0) | 0 (0) | 0 (0) | 0 (0) | 0 (0) |
| **3** | 1 | 0 (0) | 0 (0) | 0 (0) | 0 (0) | 0 (0) | 0 (0) | 9 (4) | 1 (0) | 0 (0) | 3 (2) | 0 (0) | 0 (0) | 0 (0) | 0 (0) | 0 (0) | 0 (0) | 0 (0) | 0 (0) |
| **4** | 5 | 0 (0) | 0 (0) | 0 (0) | 0 (0) | 0 (0) | 0 (0) | 21 (5) | 2 (1) | 1 (0) | 30 (8) | 5 (1) | 2 (1) | 57 (20) | 22 (7) | 43 (28) | 13 (10) | 39 (9) | 33 (7) |
| **5** | 3 | 0 (0) | 0 (0) | 0 (0) | 39 (19) | 3 (1) | 2 (1) | 0 (0) | 0 (0) | 0 (0) | 0 (0) | 0 (0) | 0 (0) | 61 (25) | 15 (7) | 7 (1) | 3 (0) | 0 (0) | 0 (0) |
| **6** | 1 | 0 (0) | 0 (0) | 0 (0) | 14 (5) | 3 (1) | 1 (0) | 27 (12) | 8 (5) | 4 (3) | 10 (2) | 3 (1) | 2 (1) | 16 (9) | 7 (3) | 0 (0) | 0 (0) | 0 (0) | 0 (0) |
| **7** | 3 | 34 (16) | 16 (8) | 11 (5) | 0 (0) | 0 (0) | 0 (0) | 0 (0) | 0 (0) | 0 (0) | 5 (2) | 0 (0) | 0 (0) | 0 (0) | 0 (0) | 0 (0) | 0 (0) | 0 (0) | 0 (0) |
| **8** | 1 | 5 (3) | 3 (1) | 0 (0) | 9 (4) | 1 (1) | 0 (0) | 4 (4) | 0 (0) | 0 (0) | 0 (0) | 0 (0) | 0 (0) | 0 (0) | 0 (0) | 0 (0) | 0 (0) | 0 (0) | 0 (0) |
| **9** | 3 | 20 (7) | 7 (3) | 3 (2) | 34 (13) | 3 (0) | 0 (0) | 15 (3) | 1 (0) | 0 (0) | 8 (2) | 2 (0) | 0 (0) | 0 (0) | 0 (0) | 0 (0) | 0 (0) | 0 (0) | 0 (0) |
| **TOTAL** | **20** | **81 (35)** | **37 (16)** | **18 (8)** | **125 (55)** | **13 (4)** | **3 (1)** | **106 (41)** | **13 (6)** | **5 (3)** | **90 (35)** | **12 (4)** | **5 (3)** | **134 (54)** | **44 (17)** | **50 (29)** | **16 (10)** | **39 (9)** | **33 (7)** |

**Supplementary Table 1 | Number of recording sessions, recorded and responsive units per patient per area.** Number of single units in parenthesis. For each area and patient, the number of recorded units per area appears under "Units". The number of responsive units during non-rivalrous condition appears under "NON-RI" – these are the MTL units that were used in the rivalry/replay analysis. The number of units that were responsive during rivalry and/or replay appears under "RI/RE". In the frontal sites no selective responses were found during the non-rivalrous conditions, therefore all recorded units were analyzed for responsiveness during rivalry and or replay. PHG = parahippocampal gyrus; EC = entorhinal cortex; AMY = amygdala; HIPP = hippocampus; ACC = anterior cingulate cortex; SMA = supplementary motor area; preSMA = pre-supplementary motor area.
